# External Ulcerative Ano-Vulvitis1Presented at the Fourteenth Annual Meeting of the Iowa State Veterinary Medical Association, Des Moines, Iowa, February 11 and 12, 1902.

**Published:** 1902-05

**Authors:** S. T. Miller

**Affiliations:** Shelby, Iowa


### EXTERNAL ULCERATIVE ANO-VULVITIS.[1]

By S. T. Miller, D.V.S.,
SHELBY, IOWA.

My attention was first called to this disease in the winter of 1897, while at Kansas City, when Dr. Steddom, with other inspectors of the Bureau of Animal Industry, was sent into Kansas

[1] Presented at the Fourteenth Annual Meeting of the Iowa State Veterinary Medical Association, Des Moines, Iowa, February 11 and 12, 1902.

to investigate a peculiar outbreak of disease among cows and heifers. On their return to Kansas City they reported a disease affecting the vulva of cows and heifers. From their report Dr. Sesco Stewart, of Kansas City, made a verbal report of the disease at a meeting of the Missouri Valley Veterinary Medical Association. The next article was that of Dr. C. Miller, of Ottumwa, which appeared in the *American Veterinary Review* for April, 1901.

My attention was next called to an outbreak in my brother-in-law's herd near Harlan, Iowa, in the winter of 1900. My brother, Dr. D. H. Miller, was called to treat that outbreak. Drs. J. I. Gibson, John J. Repp, and S. H. Johnston also visited the herd. The next outbreak occurred in my own practice about October 15, 1901. In the herd there were 23 head of cows and heifers and 4 steers. On first examination I found only two or three calves affected. In about three or four days I was surprised to find all except 1 old cow affected, including the 4 steers.

The next outbreak occurring in my own practice was about November 23, 1901. There were on the farm about 35 head of cattle, including cows, heifers, steers, and calves. About half of the number were found to be affected, but there were no steers affected in this herd.

The next case was that of 26 head in a feed yard—22 steers and 4 cows—of which 4 cows and 8 steers were affected. Some of the steers were affected badly. On the same farm there were about 25 head of pure-bred Shorthorn cattle and 50 grade stock cattle. None outside the feed yard has shown any signs of the disease.

The first noticeable symptom was serous exudate rapidly forming into a brown scab, under which was very fetid pus, with extensive inflammation. The affection usually occurred on the lower portion of the lips of the vulva in heifers and cows, and in steers around the anus or roots of the tail. The scabs which formed seemed to spread rapidly, destroying more and more of the underlying tissue and forming a thicker and thicker scab. The scab if peeled off would expose a raw surface, which would bleed very readily. In a short time a new scab would be formed.

In the herds Nos. 1 and 3, I used for treatment a wash of a strong solution of mercury bichloride to cleanse the parts, after which I applied an ointment made up as follows: iodoform, 20 grains; oil of eucalyptus, 40 minims; carbolic acid, 20 minims; petrolatum enough to make 2 ounces.

This treatment affected a very speedy and permanent cure. The animals in herd No. 2 were never treated, but are slowly recovering.

### Discussion.

Dr. Repp described the gross and microscopical morbid anatomy of the disease. He said he had made some research in connection with Dr. Miller's outbreaks Nos. 1 and 2.

Dr. Brimhall, in answer to a question, said he had not met with the disease in Minnesota. He asked if anyone had made any observation as to whether or not the disease is contagious. Dr. S. T. Miller replied that he had witnessed a case in which a bull from a healthy herd had gained access to a herd in which the disease existed, served the cows there, and soon afterward served cows in the healthy herd, yet none of the healthy cows acquired the disease.

